# M6A RNA Methylation Regulator HNRNPC Contributes to Tumorigenesis and Predicts Prognosis in Glioblastoma Multiforme

**DOI:** 10.3389/fonc.2020.536875

**Published:** 2020-10-08

**Authors:** Li-chong Wang, Shu-hui Chen, Xiao-li Shen, Dang-chi Li, Hai-yun Liu, Yu-long Ji, Min Li, Kai Yu, Huan Yang, Jun-Jun Chen, Chong-zhen Qin, Ming-ming Luo, Qian-xia Lin, Qiao-li Lv

**Affiliations:** ^1^Department of Neurosurgery, The Second Affiliated Hospital of Nanchang University, Nanchang, China; ^2^Department of Radiation Oncology, Jiangxi Cancer Hospital, Nanchang, China; ^3^Jiangxi University of Technology High School, Nanchang, China; ^4^Jiangxi University of Traditional Chinese Medicine, Nanchang, China; ^5^Jiangxi Key Laboratory of Translational Cancer Research, Jiangxi Cancer Hospital, Nanchang, China; ^6^Department of Pharmacy, The First Affiliated Hospital of Zhengzhou University, Zhengzhou, China; ^7^Jiangxi Key Laboratory of Translational Cancer Research, Department of Head and Neck Surgery, Jiangxi Cancer Hospital, Nanchang, China

**Keywords:** m6A, hnRNPC, prognosis, glioblastoma multiforme, tumorigenesis

## Abstract

Glioblastoma multiforme (GBM) is the most malignant glioma with a high death rate. N6-methyladenosine (m6A) RNA methylation plays an increasingly important role in tumors. The current study aimed to determine the function of the regulators of m6A RNA methylation in GBM. We evaluated the difference, interaction, and correlation of these regulators with TCGA database. *HNRNPC, WTAP, YTHDF2* and, *YTHDF1* were significantly upregulated in GBM. To explore the expression characteristics of regulators in GBM, we defined two subgroups through consensus cluster. *HNRNPC*, WTAP, and YTHDF2 were significantly upregulated in the cluster2 which had a good overall survival (OS). To investigate the prognostic value of regulators, we used lasso cox regression algorithm to screen an independent prognostic risk characteristic based on the expression of *HNRNPC, ZC3H13*, and *YTHDF2*. The prognostic feature between the low and high-risk groups was significantly different (*P* < 0.05), which could predict significance of prognosis (area under the curve (AUC) = 0.819). Moreover, we used western blot, RT-PCR, and immunohistochemical staining to verify the expression of *HNRNPC* was associated with malignancy and development of gliomas. Similarly, the high expression of *HNRNPC* had a good prognosis. In conclusion, *HNRNPC* is a vital participant in the malignant progression of GBM and might be valuable for prognosis.

## Introduction

Gliomas are the most common primary malignant tumors of the central nervous system (CNS). Glioblastoma multiforme (GBM), a class IV neoplasm with astrocytic differentiation, is the most aggressive and lethal glioma (1). GBM is characterized by a poor prognosis, and people who developed a GBM had a median survival rate of < ~1 year and a high death rate (2). To date, nearly 150 types of posttranscriptional modifications have been discovered in RNA among all living organisms (3). N6-methyladenosine (m6A) RNA methylation is the most prevalent post-transcriptional modification mechanism in humans (4, 5). Its function and mechanism have not been investigated until recently, since it was discovered in the 1970s (6). The biological function of m6A modification is coordinated by multiple writers like methyltransferase-like enzymes *METTL3* and *METTL14*, Wilms tumor 1-associated protein (*WTAP*), *KIAA1429*, RNA binding motif protein 15 (*RBM15*), and zinc finger CCCH-type containing protein 13 (*ZC3H13*), erasers like fat mass- and obesity-associated protein (*FTO*) and α-ketoglutarate-dependent dioxygenase AlkB homolog 5 (*ALKBH5*), and readers like YTH domain-containing 1 (*YTHDC1*), YTH domain-containing 2 (*YTHDC2*), YTH N6-methyl-adenosine RNA binding protein 1 (*YTHDF1*), YTH N6-methyladenosine RNA binding protein 2 (*YTHDF2*), and heterogeneous nuclear ribonucleoprotein C (*HNRNPC*) (7–12). The discovery of regulators increased our perception of the function of the m6A modification. M6A modification in cancer is a double-edged sword: it inhibits tumor progression in some cancers and promotes tumor progression in other cancers. It exerts vital functions in mammals, including embryonic development, neurogenesis, stress responses, sex determination, and tumorigenesis (13, 14). Studies have revealed that changes in m6A levels in glioblastoma stem cell-like cells (GSCs) seriously affect tumor growth, self-renewal, and development (15). However, the literature lacks a comprehensive analysis of the expression levels, prognostic values, and functions of m6A RNA methylation regulators in GBM.

In this study, we downloaded the original RNA-seq GBM dataset from The Cancer Genome Atlas (TCGA) (*n* = 174), a public database for archiving and querying cancer data. We observed the expression of the regulators in the dataset and found that *HNRNPC* not only played a vital role in the differentially expressed genes, but acted as a signature that could be designed to stratify the prognosis of GBM. In addition, western blot, RT-PCR, and immunohistochemical staining were performed showing that *HNRNPC* might be associated with malignant progression of GBM and might predict a good prognosis.

## Materials and Methods

### Data Acquisition

Raw RNA-seq data (FPKM files) and clinical data on GBM were extracted from the TCGA data portal (https://tcga-data.nci.nih.gov/tcga/: accessed September 2019). These data on GBM tissue samples (*n* = 169) and normal brain tissue samples (*n* = 5) were downloaded from the TCGA.

### Identification of Genes Related to m6A

The thirteen m6A RNA methylation regulators (*METTL3, METTL14, WTAP, KIAA1429, RBM15, ZC3H13, YTHDC1, YTHDC2, YTHDF1, YTHDF2, HNRNPC, FTO*, and *ALKBH*5) were collated based on a previously published article (7–12). Then, we systematically compared the expression levels of these m6A RNA methylation regulators between GBM and normal brain tissues and used the Wilcoxon signed-rank test to perform a differential expression analysis. Heatmaps and violin charts were generated with R 3.6.1 (https://www.r-project.org/).

### Bioinformatic Analysis

Interactions and Gene Ontology (GO) analysis of the m6A RNA methylation regulators were performed using the STRING database (http://www.string-db.org/). GEPIA (16) (http://gepia.cancerpku.cn/) is a web server for analyzing the RNA sequencing expression data of 9,736 tumor samples and 8,587 normal samples from the TCGA and the Genotype-Tissue Expression (GTEx) project using a standard processing pipeline. |log_2_FC| is defined as median of the expression and the cut point of |log_2_FC| is 1, the cutoff of *p*-value is 0.01.

The relation among the regulators was calculated by Pearson's correlation based on gene expression. To explore the function of m6A RNA methylation regulators in GBM, we clustered gliomas into different groups with “ConsensusClusterPlus” (50 iterations, resample rate of 80%, and Pearson's correlation http://www.bioconductor.org/). Then, principal component analysis (PCA) was performed with R 3.6.1 to examine gene expression patterns in different glioma groups.

To determine the prognostic value of m6A RNA methylation regulators, we performed univariate Cox regression analyses of their expression in the TCGA. Then, all regulators were selected for the functional analysis and development of a potential risk signature with the least absolute shrinkage and selection operator (LASSO) Cox regression algorithm. Finally, three genes and their coefficients were selected with the minimum criteria, choosing the best penalty parameter λ associated with the smallest 10-fold cross-validation within the training set. The risk score for the signature was evaluated by using the following formula:

$$\begin{array}{l} Riskscore=\overset{n}{\underset{i=1}{\sum }}Coef_{i}*x_{i} \end{array}$$

where *Coef*_*i*_ and *x*_*i*_ are respectively the coefficient and the *z*-score-transformed relative expression value of each selected gene. This formula was used to calculate the risk score for each GBM patient in the TCGA dataset.

### Patients Samples

The 116 primary glioma samples and clinical information were acquired from postoperative patients, who undergone surgical treatment first time without chemotherapy or radiotherapy between 2013 June and 2014 January at Xiangya Hospital of Central South University. Eighteen normal brain tissues from patients with cerebral trauma surgery were collected as controls. The informed consents were provided to all patients or their family members. All the participants agreed the informed consents. All of samples were instantly frozen in liquid nitrogen and stored at −80°C until the extraction of total RNA. Because of multiple parameters like the lost to follow-up, only 61 of the patients experienced a follow-up period lasting 5 years since the surgery. This study was agreed by Central South University Xiang ya Hospital Medical Ethics Committee.

### RNA Extraction and Real-Time PCR

According to manufacturer's instructions, total RNA was extracted from glioma and normal samples using TRIzol reagent (Invitrogen, Carlsbad, CA, USA) and cDNA was synthesized from 1 μg of total RNA using a Primescript™ RT reagent kit with gDNA eraser (TakaRa, Japan). RT-PCR was carried out by using SYBR Premix Dimer Eraser TM (Takara, Dalian, China) to detect the expression of mRNA *HNRNPC*, with *GAPDH* as a normalizing control. The following primers were used: *HNRNPC* F: 5′-CCTTACCATCAAACACGATGGC-3′, R: 5′-ACTTCGAAAAGATTGCCTCCACA-3′. GAPDH:F: 5′-CCCATCACCATCTTCCAGGAG-3′, R: 5′-GTTGTCATGGATGACCTTGGC-3′.

### Western Blot

Protein concentrations were estimated using the BCA protein assay. Total proteins were extracted using cold RIPA buffer with Phenylmethanesulfonyl fluoride (Beyotime, China) and phosphatase Inhibitor Cocktail 2 (sigma, USA) for 30 min on ice, and centrifuged at 12,000 × g for 15 min at 4°C. Antibodies against *HNRNPC* were obtained from Proteintech. Antibody to β-actin was used as a normalizing control.

### Immunohistochemistry

The expression of *HNRNPC* was detected by IHC in 25 cases of glioma tissues. The 5-μm-thick glioma tissue sections were dewaxed in xylene, rehydrated through decreasing concentrations of ethanol and washed in distilled water. According to the standard protocols, sections were processed and stained with hematoxylin and eosin (H&E) and diaminobezidine. At last, sections were dehydrated through increasing concentrations of ethanol and xylene to the transparent state, and sealed with neutral gum. Primary antibody was diluted at 1:100 according to the manufacturer's instructions. *HNRNPC* positive cells in microscopic fields at ×400 and ×100 were observed and the ICH results were assessed by two pathologists, respectively.

### Statistical Analysis

Wilcoxon rank-sum and signed-rank tests were texted to evaluate the expression levels of m6A RNA methylation regulators in GBM for different groups. Univariate and multivariate Cox regression analyses were tested to determine the prognostic value of the regulators. Based on the consensus expression of thirteen regulators, 169 patients with GBM were divided into two subgroups by using consensus cluster and divided into low-risk and high-risk groups by using the median risk score (originated from the risk signature) as a break point. Chi-square tests were performed to compare the characteristics of clinical features (*n* = 158) such as gender, race, age, IDH, and p53 mutant information, and survival time between the two risk groups and two clusters. The receiver operating characteristic (ROC) curves were used to show the prediction efficiency of the risk model for 5-year survival in clusters 1 and 2. The Kaplan–Meier method was used to compare the overall survival (OS) rates of patients in the high- and low-risk groups, while the log-rank test was tested to compare the survival distributions between two groups. The Cut-off point was used the mean of the expression of HNRNPC. All statistical analyses were conducted using R 3.6.1. Experimental data analysis was performed with the Graphpad Prism 8.0 (GraphPad, San Diego, CA, USA). The *t*-test was performed to detected differential expression of *HNRNPC* between glioma in different grades and normal brain tissues. The error bars in bar graphs were represented the mean ± SD (Standard Deviation). A *p*-value of < 0.05 was considered statistically significant.

## Results

### m6A RNA Methylation Regulators Are Significantly Different Between Tumor and Normal Brain Tissues

**Figure 1:**
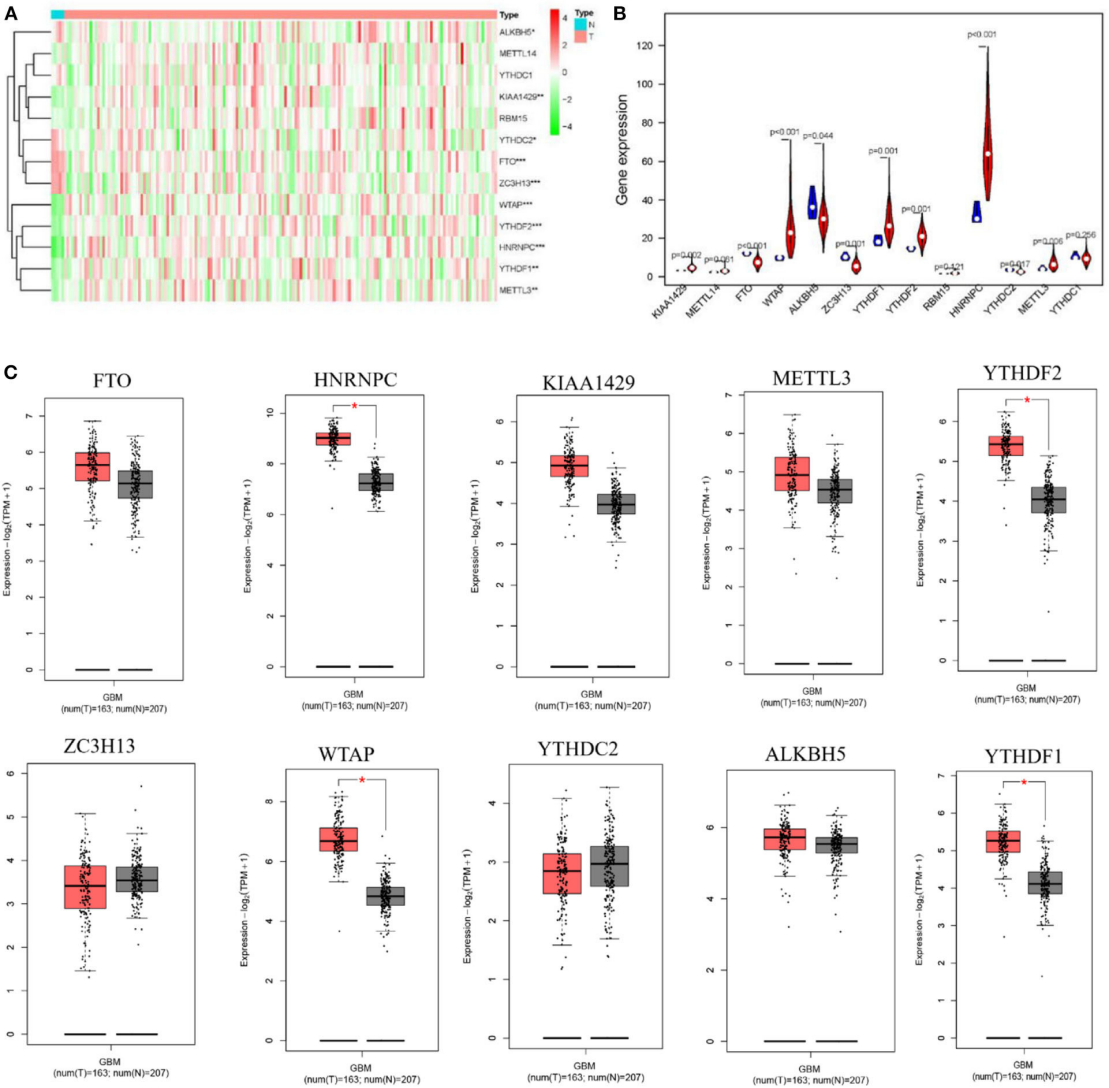
Expression of m6A RNA methylation regulators in GBM and normal tissue. **(A,B)** The heatmap and the violin diagram showed the m6A RNA methylation regulators in GBM and normal tissues. GBM was marked with red and normal tissues were marked with blue, position of white spots on the way represented the median value of expression. **(C)** The boxplots structured by GEPIA showed a difference between GBM and large number of normal brain tissues. The num(T) represented the number of tumors which were marked with red, the num(N) represented the number of normal tissues which were marked with blue (*p* < 0.001 noted with ***p < 0.01 noted with **p < 0.05 noted with *).

To study the functions of the m6A RNA methylation regulators in the tumorigenesis and progression of GBM, differences between tumor and normal brain tissues were investigated and presented as a heatmap (Figure 1A, ^*^p < 0.05, ^**^p < 0.01, and ^***^p < 0.001), showing that almost all the regulators were highly related to the oncogenesis and development of GBM. The differences between genes were presented as a violin plot (Figure 1B). The expression levels of *METTL3, WTAP, KIAA1429, ZC3H13, YTHDC2, YTHDF1, YTHDF2, HNRNPC, FTO*, and *ALKBH5* were significantly different between GBM and normal tissues. Considering the small number of normal brain tissues in the TCGA, we investigated the expression of the m6A regulators on the online web server GEPIA, which contains large dataset of 9,736 tumors and 8,587 normal samples from the TCGA and the GTEx project, respectively (Figure 1C, ^*^p < 0.05). A comparison of all results showed that the *HNRNPC, WTAP, YTHDF2*, and *YTHDF1* were up-regulated in tumor samples and might influence the tumorigenesis of GBM.

### Interactions and Correlations Among the m6A RNA Methylation Regulators in GBM

**Figure 2:**
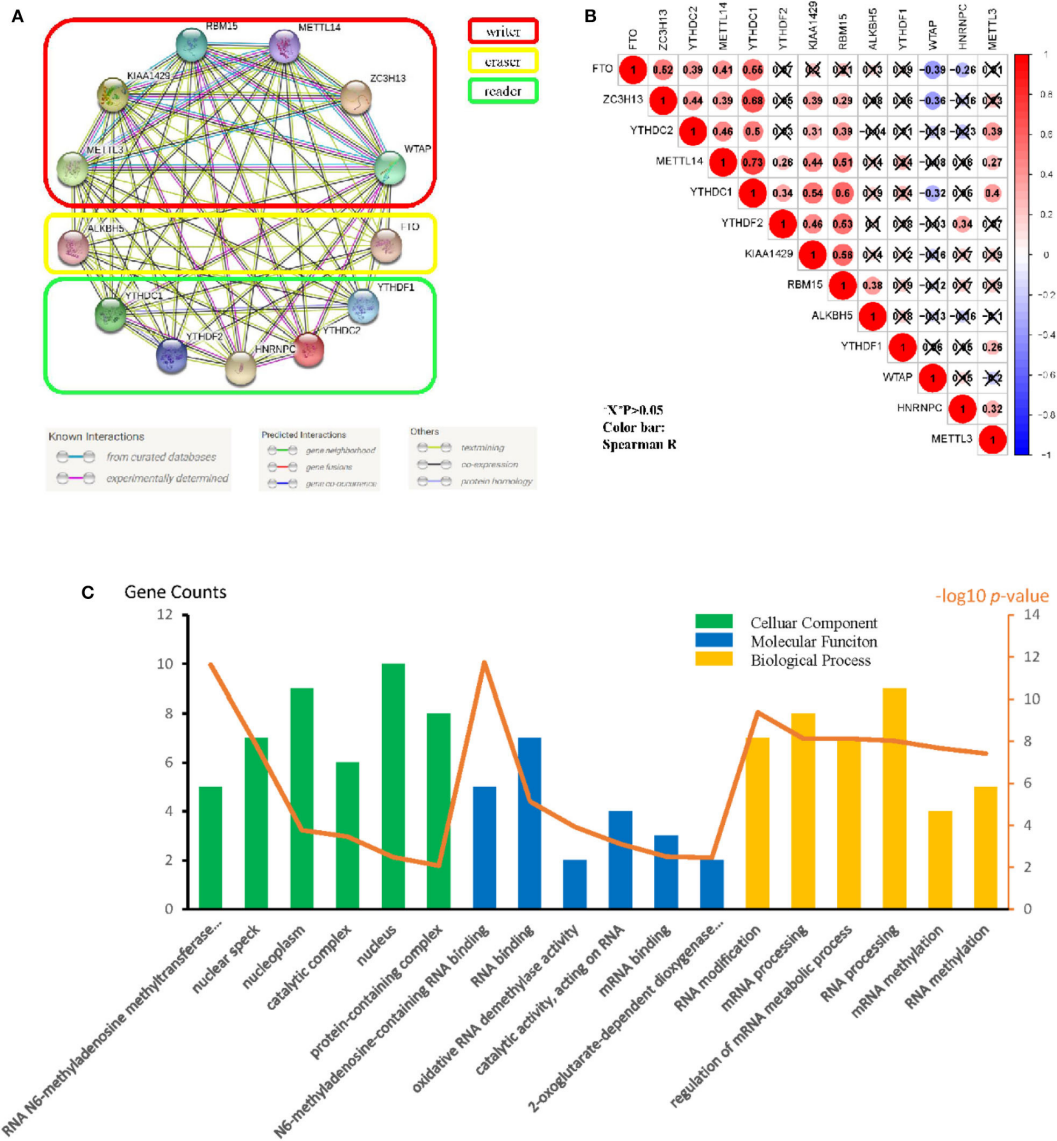
Protein–protein interaction (PPI), go ontology, and correlation of the m6A RNA methylation regulators. **(A)** M6A modification-related Protein–protein interactions among the 13 m6A RNA methylation regulators. **(B)** Spearman correlation analysis of the 13 m6A methylation modification regulators. **(C)** Gene ontology (GO) analysis classified regulators into BP (Biological Process), CC (cellular Component), and MF (Molecular Function) groups.

Originated from the STRING online database (https://string-db.org/) and all m6A methylation regulators were filtered into the PPI network complex (Figure 2A). The correlations among the regulators were analyzed (Figure 2B). WTAP and FTO appeared to have a negative correlation, while *METTL14* and *YTHDC1* showed the most positive correlation. We also identified GO terms and Kyoto Encyclopedia of Genes and Genomes (KEGG) pathways and classified them into three functional categories: biological process (BP), cellular component (CC), and molecular function (MF). The top three enriched BP terms were “RNA modification,” “mRNA processing,” and “regulation of mRNA metabolic process.” For CCs, the top three terms were “RNA N6-methyladenosine methyltransferase complex,” “nuclear speck,” and “nucleoplasm.” The top three enriched MF terms were “N6-methyladenosine-containing RNA binding,” “RNA binding,” and “oxidative RNA demethylase activity.” All the results were shown in Figure 2C. The pathway enrichment analysis showed that the genes were associated with the reversal of alkylation damage by DNA dioxygenases and processing of the capped intron-containing pre-mRNA signaling pathway (Supplementary Table 1). These findings showed that the regulators had a broad connection to the RNA modification and cancer processes.

### Two GBM Subgroups Were Clustered by Distinct Clinical Survival Times

**Figure 3:**
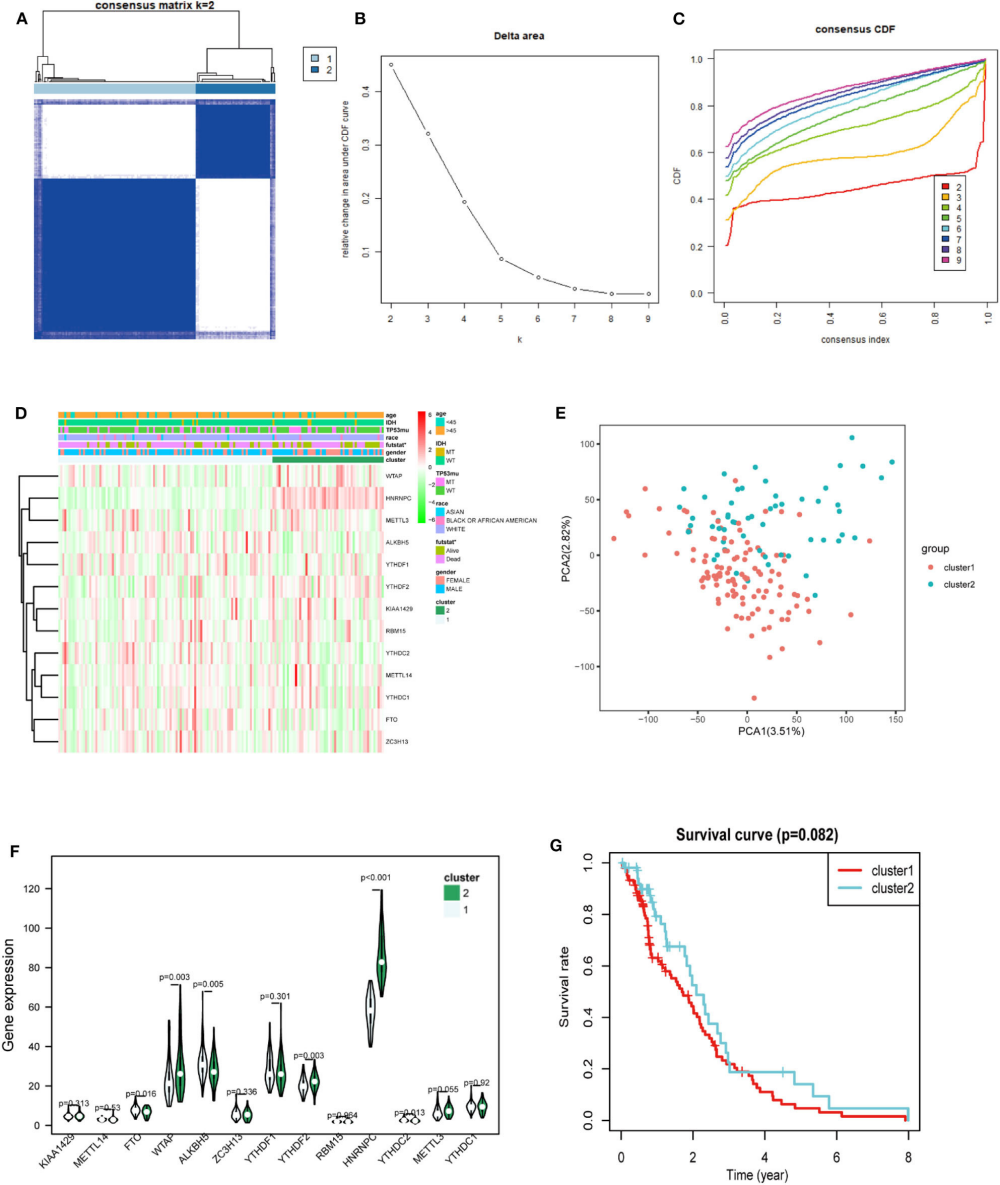
Differential expression and overall survival of GBM in the two subgroups. **(A)** The TCGA GBM cohort was divided into two clusters when *k* = 2. **(B)** Relative change in area under CDF curve for *k* = 2–9. **(C)** Consensus clustering cumulative distribution function (CDF) for *k* = 2–9. **(D)** The heatmap showed the relation between the two clusters and the clinical characteristic. **(E)** PCA (Principal component analysis) of the total RNA expression profile in GBM cluster1 was marked with red. **(F)** The violin chart of the two clusters (1 and 2) defined by the m6A RNA methylation regulators consensus expression (green represented the cluster 2) and position of white spots on the way represented the median value of expression. **(G)** Kaplan–Meier overall survival (OS) curves for GBM patients (*p* < 0.05 noted with *).

Considering the dramatic imbalance in the numbers of GBM (*n* = 169) and normal brain tissue samples (*n* = 5) and the in-depth knowledge of GBM, we identified two new clusters of GBM based on the expression of all of the genes related to m6A RNA methylation (*k* = 2, which appeared to fit with the selection based on clustering stability increasing from *k* = 2 to 9 in the TCGA dataset) (Figures 3A–C). However, 159 of 169 gliomas clustered into one of the two subgroups in the TCGA dataset. Different clinical characteristics and the expression of m6A regulators between the two clusters (Figure 3D) showed that the different clusters are related to the survival status but the age, race, gender, IDH, and p53 mutant and *HNRNPC, ALKBH5, WTAP, YTHDF2, YTHDC2*, and *FTO* were markedly different between the two groups (Figure 3F). *HNRNPC, WTAP*, and *YTHDF2* were upregulated in cluster 2, while *ALKBH5, YTHDC2*, and *FTO* were down-regulated in cluster 2. Principal component analysis (PCA) was performed to compare the transcriptional profiles between the cluster 1 (*n* = 105) and cluster 2 (*n* = 54) subgroups. There was a clear distinction between them (Figure 3E). Then, we compared the clinical survival outcomes of these two subgroups (cluster 1 and 2) clustered by *k* = 2. Interestingly, cluster 2 seemed to have a good survival trend (Figure 3G). Therefore, we believed that *HNRNPC, WTAP*, and *YTHDF2* might be predictors of a high survival rate.

### Functional Annotation of GBM in Clusters 1 and 2

**Figure 4:**
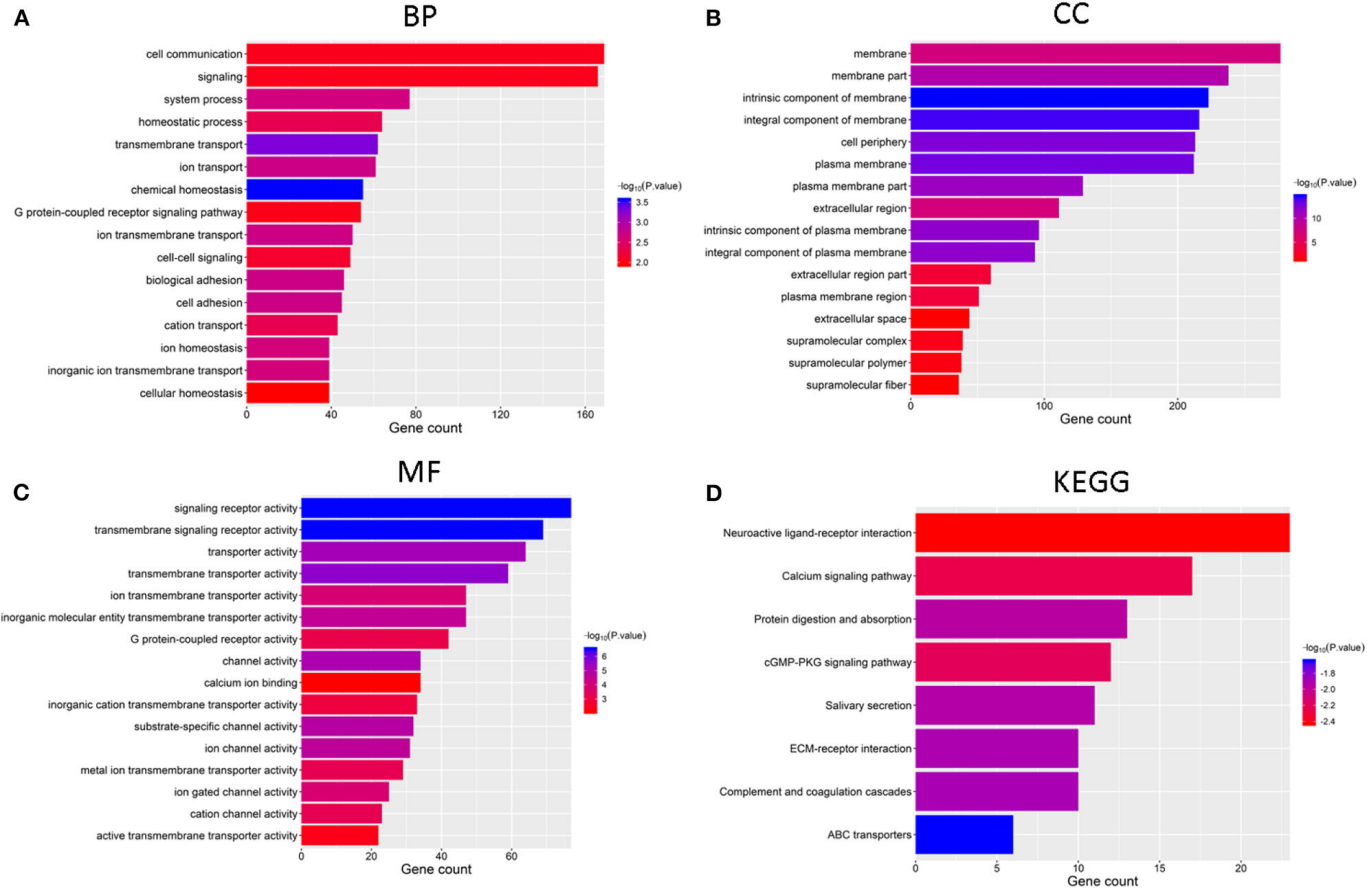
Functional annotation of differential expression of GBM in the two subgroups, Functional annotation of the genes with lower expression in the cluster2 subgroup using GO terms of biological processes **(A)**, cellular component **(B)**, molecular function **(C)**, and KEGG pathway **(D)**.

We identified genes that were significantly changed (|log_2_-fold-change| > 1 and normalized *p* < 0.05) in the cluster 2 subgroup and then annotated their function using GO and pathway analyses. The results indicated that the downregulated genes in cluster2 are enriched in BPs including “chemical homeostasis,” “transmembrane transport,” and “ion transport” (Figure 4A), CCs including “intrinsic component of membrane,” “integral component of membrane,” and “plasma membrane” (Figure 4B) and MFs including “transmembrane signaling receptor activity,” “signaling receptor activity,” and “transmembrane transporter activity” (Figure 4C). Additionally, genes involved in KEGG pathways were enriched in “cGMP-PKG signaling pathway,” “neuroactive ligand-receptor interaction,” and “calcium signaling pathway” (Figure 4D). The upregulated genes were enriched in intracellular protein transport and ribosome (Supplementary Figure 1). All these findings indicate that the cluster groups divided by the expression of regulators are associated with the development of cancer.

### Prognostic Indices of m6A RNA Methylation Regulators and a Risk Signature Built Using Three Regulators

**Figure 5:**
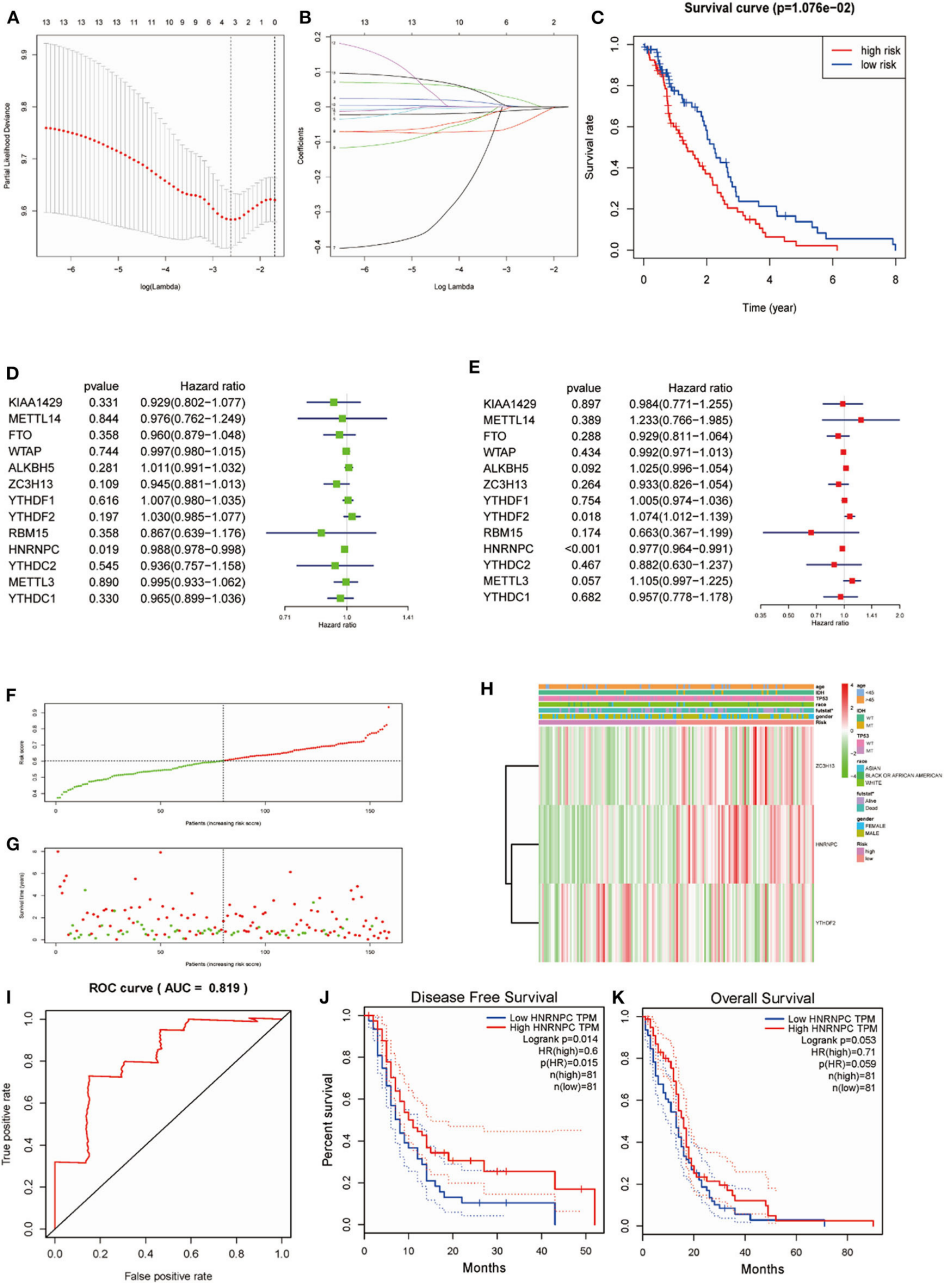
Risk signature with three m6A RNA methylation regulators and *HNRNPC* was the key gene in predicting the prognosis. **(A,B)** The process of building the signature containing 13 m6A RNA methylation regulators. **(C)** Kaplan–Meier overall survival (OS) curves for GBM patients in the TCGA datasets assigned to high- and low-risk groups based on the risk score (*p* = 1.076e−02). **(F)** The distributions of risk scores. **(D)** The hazard ratios (HR), 95% confidence intervals were calculated by univariate Cox regression. **(E)** The hazard ratios (HR), 95% confidence intervals were calculated by multivariate Cox regression. **(G)** The distributions of risk scores and OS status. The green and red dots indicated the alive and dead status, respectively. **(H)** Heatmap and clinical features by the m6A RNA methylation regulators risk signature. **(I)** ROC curves showed the predictive efficiency of the risk signature on the 5-year survival rate (AUC = 81.9%). **(J,K)** Kaplan–Meier analysis was performed to investigate the association of *HNRNPC* expression level with the Overall survival (OS) and disease-free survival (DFS).

Considering the prognostic indices, we sought to investigate the prognostic role of m6A RNA methylation regulators in GBM. We performed univariate and multivariate Cox regression analysis on the expression levels in the TCGA dataset (Figures 5D,E). The results indicated that *HNRNPC* might be a protective gene that is significantly related to OS, with a hazard ratio (HR) < 1 (*p* < 0.05). The remaining tested genes were not (*p* > 0.05). To better predict the clinical outcomes of GBM with m6A RNA methylation regulators, the LASSO Cox regression algorithm was applied to thirteen genes. Three genes (*HNRNPC, ZC3H13*, and *YTHDF*2) were used to build a risk signature based on minimum criteria, and the coefficients obtained from the LASSO algorithm were used to calculate the risk score for the TCGA dataset (Figures 5A,B). We separated patients with GBM from the TCGA (*n* = 159) dataset into low-risk (*n* = 80) and high-risk (*n* = 79) groups and observed that GBM patients in the low-risk group had significantly longer OS than those in the high-risk group (*p* = 1.076e−02) (Figure 5C), showing that the model could predict prognosis well.

**Figure 6:**
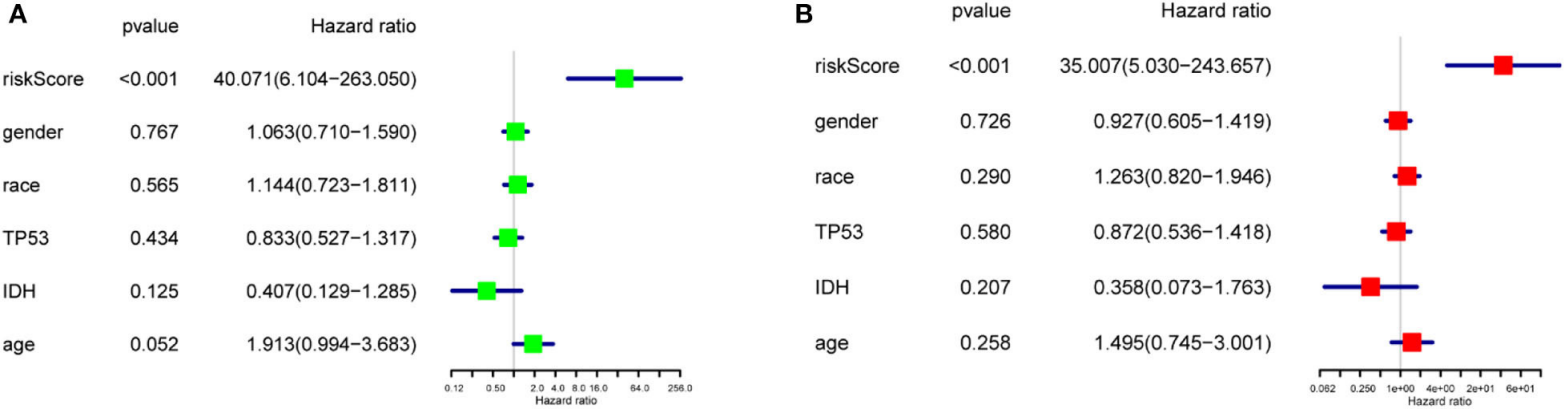
The relationship between the clinical characters and the prognosis by univariate and multivariate Cox regression in TCGA database **(A)** The hazard ratios (HR), 95% confidence intervals were calculated by univariate Cox regression. **(B)** The hazard ratios (HR), 95% confidence intervals were calculated by multivariate Cox regression.

To study the prognostic value of the model. Figure 5F showed the distributions of the three gene signature-based risk scores. The distributions of risk scores and OS status were displayed in Figure 5G, and the heatmap showed the expression levels of the m6A regulators in the low- and high-risk groups. Different clinical characteristics and the expression of m6A regulators between the two groups (Figure 5H, *n* = 158) showed that the different group are related to the survival status but the age, race, gender, IDH, and p53 mutant. As shown in Figure 5I, the ROC curve revealed that the risk score could perfectly predict the five-year survival rates for GBM patients (AUC = 81.9%). We also calculated the OS and disease-free survival (DFS) rates of *HNRNPC* in the TCGA GBM dataset by GEPIA (Figures 5J,K). We observed that high expression of HNRNPC was associated with a good prognosis. Also, we identified the relationship between the clinical and the prognosis in TCGA database by univariate and multivariate Cox regression. As is shown in the Figures 6A,B, only the risk-score are associated to the prognosis. These results confirmed that the risk score originating from the selected m6A RNA methylation regulators could predict prognosis in GBM patients. And *HNRNPC* might be the key gene for prognostic value.

### *HNRNPC* Correlates With Cancer Progression and Development in Human Glioma Tissue

**Figure 7:**
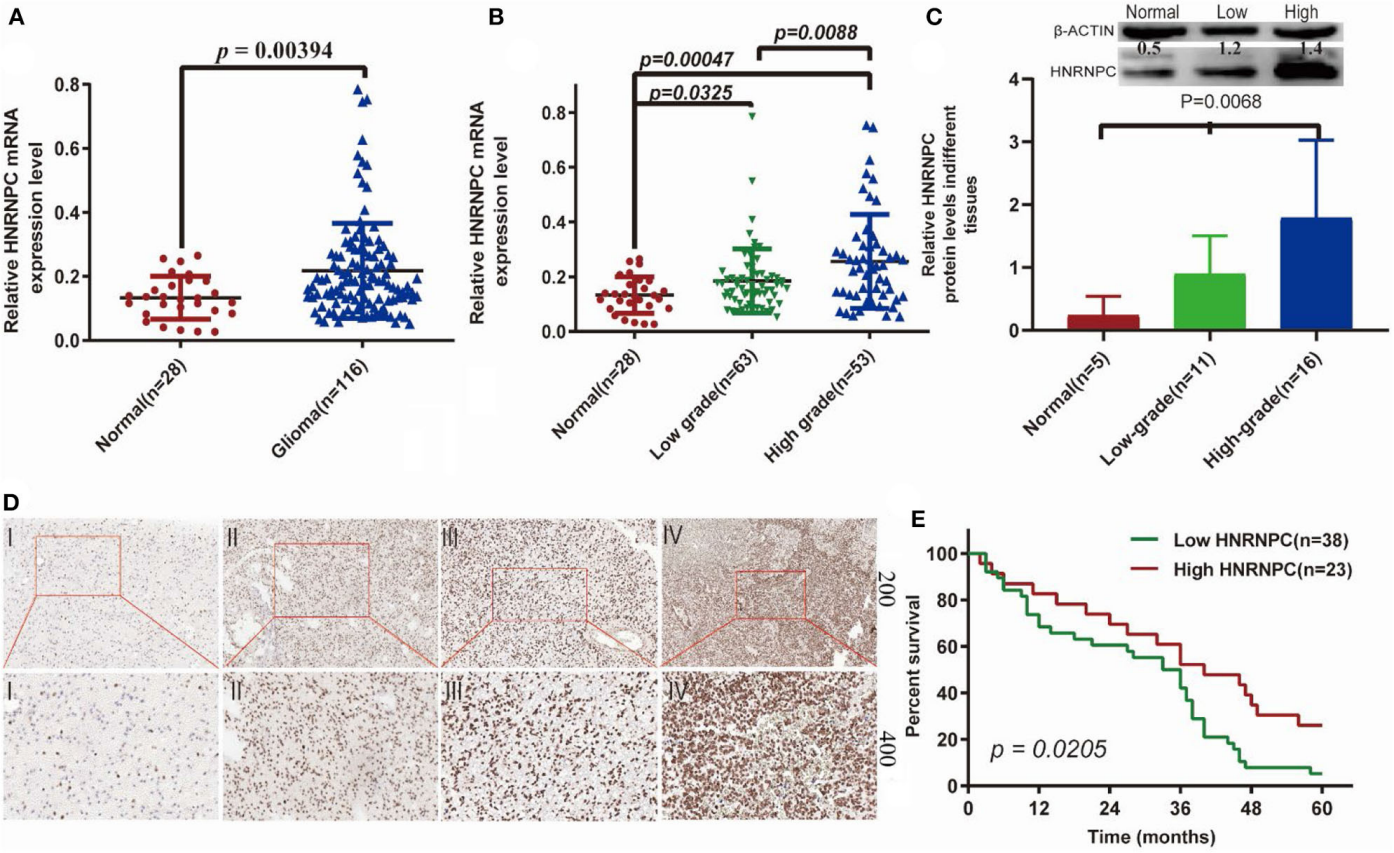
The expression of *HNRNPC* was associated with histological malignancy and clinical outcome in human gliomas. **(A)** The mRNA expression levels of *HNRNPC* in glioma and normal brain tissues were detected by RT-PCR assays. **(B)** *HNRNPC* mRNA expression levels in different glioma tissues. **(C)** The protein levels of *HNRNPC* in normal brain tissues and different grades of glioma were evaluated **(D)** The expression of *HNRNPC* was examined by immunohistochemistry staining in glioma tissues. **(E)** Kaplan–Meier analysis was evaluated to check the association of *HNRNPC* expression level with the Overall survival (OS) time in 61 cases of glioma patients (*p* = 0.0268).

To verify whether *HNRNPC* played an important role in the development and progression of glioma, we firstly evaluated its mRNA expression level in glioma (*n* = 116) and normal brain (*n* = 28) tissues by RT-PCR, which indicated that *HNRNPC* expression was upregulated in glioma tissues compared with normal brain tissues (Figure 7A, *p* < 0.01). As shown is Additionally, we observed a relationship between the *HNRNPC* expression level and histological malignancy in tissues with different grades. As shown in Figure 7B, with the increase in *HNRNPC* expression, the malignancy of glioma showed an increasing tendency (*p* < 0.01). We examined the relationship of *HNRNPC* levels with overall survival (OS) rates through Kaplan–Meier analysis and log-rank test in 61 glioma cases which were collected between 2013 and 2014 with a 5-years followed up information, showing that the high expression of *HNRNPC* seems to be correlated with a good prognosis (Figure 7E, Hazard Ratio (HR) = 1.892, 95%Confidence Interval (CI) = 1.103–3.244, and *p* = 0.0205). All the results verified our prediction that *HNRNPC* might be a key gene in progression and development of GBM. The upregulation of *HNRNPC* in glioma tissues was further evaluated by Western blot (WB) and immunohistochemical (IHC) staining. For WB, we used 5 normal brain tissues, 11 low-grade (grades I-II) malignant glioma tissues, and 16 high-grade (grades III-IV) malignant glioma tissues and found that the protein level of *HNRNPC* was dramatically higher in high-grade and low-grade malignant glioma tissues than in normal brain tissues (Figure 7C, *P* < 0.05). *HNRNPC* expression was further examined by IHC staining in another 25 glioma tissues. We investigated the positive immunoreactivity of *HNRNPC* in different grades of human glioma tissues, as shown in Figure 7D, which indicated that the expression of *HNRNPC* was significantly related to the grades.

### Upregulation of *HNRNPC* Might Be Associated With Good Prognosis of Glioma Patients

To determine the clinical significance of *HNRNPC* in glioma, we examined the correlation of *HNRNPC* expression with clinicopathological parameters (Table 1), observing that increased *HNRNPC* expression level was significantly associated with tumor grade of glioma (*p* = 0.0001), rather than age, gender, and tumor location. All the results implied that *HNRNPC* might play a vital role in glioma progression.

**Table 1:** Correlation between HNRNPC expression and clinicopathological features of glioma patients.

**Clinical characteristic**	**No. of patients**	**No. of patients**		***P*-value**
		**High expression (42)**	**Low expression (74)**	
**Age (years)**				
<45	51	15	36	0.1774
≥45	65	27	38	
**Sex**				
Male	76	29	47	0.5467
Female	40	13	27	
**Clinical stage**				
Low grades I–II	63	13	50	0.0001^*^
High grades III–IV	53	29	24	
**Tumor location**				
Frontal	43	16	27	0.4760
Parietal	8	2	6	
Occipital	15	6	9	
Temporal	19	4	15	
Others	31	14	17	

* *The values had statistically significant differences (P < 0.05)*.

To further elucidate the correlation of HNRNPC expression with prognosis of glioma patients, univariate analysis demonstrated that the clinical stage (HR = 2.508, 95% CI = 1.450–4.338, and *p* = 0.001) and HNRNPC expression level (HR = 0.510, 95% CI = 0.282–0.923, and *p* = 0.026) were associated with prognosis. Multivariable Cox regression analysis confirmed that the clinical stage (HR = 2.688, 95% CI = 1.359–5.314, and *p* = 0.004) and the highly expressed HNRNPC (HR = 0.520, 95% CI = 0.283–0.955, and *p* = 0.035) were independent prognostic factors for OS of glioma patients (Table 2). These results suggested that upregulated HNRNPC seemed to be a prognostic marker for glioma patients.

**Table 2:** Univariate and multivariate analyses of OS in 61 glioma patients by Cox regression analysis.

	**Univariate analysis**		**Multivariate analysis**	
Variable	HR (95% CI)	*P*-value	HR (95% CI)	*P*-value
Age (<45 vs. ≥45 years)	1.249 (0.716–2.180)	0.434	1.470 (0.724–2.985)	0.287
Sex (female vs. male)	0.735 (0.418–1.290)	0.283	0.791 (0.389–1.609)	0.517
Clinical stage (III–IV vs. I–II)	2.508 (1.450–4.338)	0.001^*^	2.688 (1.359–5.314)	0.004^*^
Tumor location		0.298		0.875
Parietal vs. frontal	1.465 (0.745–2.579)	0.268	1.006 (0.457–2.212)	0.988
Occipital vs. frontal	1.769 (0.574–5.452)	0.320	0.756 (0.210–2.725)	0.668
Temporal vs. frontal	0.741 (0.266–2.063)	0.566	0.648 (0.217–1.934)	0.437
Others vs. frontal	0.700 (0.292–1.681)	0.425	0.735 (0.280–1.924)	0.530
HNRNPC expression (high vs. low)	0.510 (0.282–0.923)	0.026^*^	0.520 (0.283–0.955)	0.035^*^

* *The values had statistically significant differences. The bold values represent a significant P-value which is less than 0.05*.

## Discussion

At present, a low cure rate is a major challenge to GBM patients. In clinical practice, current common treatment options for GBM include maximal tumor excision, radiation therapy, and chemotherapy with common antineoplastic agents (such as Temozolomide) (17). Despite advances in GBM diagnosis and treatments, its poor prognosis remains a difficult challenge due to the highly aggressive and extremely infiltrative features of GBM (18, 19). Therefore, it is urgent to identify prognostic biomarkers for the tumorigenesis and development of GBM in patients who might benefit from curative therapy. With in-depth studies, epigenetic processes have become more valuable for the diagnosis and therapy of cancer. As a player in the epigenetic process, m6A methylation has a complex function in cancer. Studies have shown that m6A-regulating proteins could induce oncogene expression, cancer cell proliferation, survival, and tumor initiation and progression (20).

This study first showed the association between m6A regulators and prognosis and the relationship and function of the regulators. Based on the TCGA data, the expression levels of regulators related to m6A RNA methylation were analyzed in GBM (*n* = 169) and normal (*n* = 5) tissues. Ten of the thirteen m6A regulators, namely, *METTL3, WTAP, KIAA1429, ZC3H13, YTHDC2, YTHDF1, YTHDF2, HNRNPC, FTO*, and *ALKBH5* showed a significant difference in GBM. Then, we filtered these regulators in GBM (*n* = 163) and normal brain (*n* = 207) tissues with GEPIA. Last, we found that *HNRNPC, WTAP, YTHDF2*, and *YTHDF1* were significantly upregulated, indicating that these genes might be associated with the progression of GBM. In present study, the relationship among regulators was also revealed. The expression of WTAP was negatively associated with the expression of FTO, and the expression of *METTL14* and *YTHDC1* showed the most positive correlation. The functions of the m6A regulators were enriched in mRNA processes and RNA binding, which indicated that they were related to cancer progression. Regarding one of the enriched KEGG pathways, the “processing of capped intron-containing pre-mRNA signaling pathway” might promote the survival of malignant cells following therapy (21, 22). Among the regulators, *METTL3* has been discovered to regulate cell proliferation, tumor growth, self-renewal, and development in glioma (23). *METTL14* has been reported as a tumor suppressor gene in GBM, and the erasers FTO and ALKBH5 are oncogenes in glioma (24–26).

Furthermore, to find key m6A genes related to OS and process in GBM, two GBM subgroups were constructed by consensus clustering based on the expression of m6A RNA methylation regulators. The results showed *HNRNPC, WTAP*, and *YTHDF2* were upregulated in cluster 2, while *ALKBH5, YTHDC2*, and *FTO* were downregulated in cluster 2. Differences in OS between the two subgroups were investigated, which indicated that the levels of m6A regulators were associated with the prognosis of GBM. Although no significant difference in OS was observed, a trend between the two clusters could also be discovered. Cluster 2 was associated with a good prognosis. We speculate that statistical significance might be detected by increasing the sample numbers. In addition, GO and KEGG pathway analyses were also conducted between the two clusters. Most of the enriched functions of differentially expressed genes (DEGs) identified by the two clusters were related to the cancer process. Regarding the KEGG pathways, “cGMP-PKG signaling pathway” and “calcium signaling pathway” were enriched. The calcium signaling pathway acts in various biological processes, such as the cell cycle and survival (27, 28). The cGMP-PKG signaling pathway plays an important role in tumor cell proliferation and apoptosis and prevents the progression of colon cancer (29–31). These findings indicated these regulators were related to the initiation and development of GBM and *HNRNPC, WTAP*, and *YTHDF*2 might be associated with a good prognosis.

In this study, the prognostic value of m6A regulators was subsequently evaluated. A univariate Cox analysis was performed to predict the prognostic value of the m6A regulators. *HNRNPC* showed a good performance (*p* < 0.05) for predicting the clinical outcome of GBM. In addition, a prognostic model was constructed using the three genes (*ZC3H13, HNRNPC*, and *YTHDF2*) identified by LASSO regression, which stratified the OS of patients with gliomas into high- and low-risk groups. The high-risk group suffered a poorer clinical outcome than the low-risk group. Furthermore, this model could predict the prognosis of patients well and be used to provide novel ideas for clinical applications in GBM. The prognostic model shows that the expression level of *ZC3H13* was positively associated with the prognosis of GBM. *ZC3H13* might act as a tumor suppressor gene, as we would expect in GBM. *ZC3H13* could suppress proliferation and invasion in colorectal cancer and regulate mouse embryonic stem cell self-renewal (32, 33). Our prognostic model revealed that the expression level of *HNRNPC* was positively associated with the prognosis of GBM. Heterogeneous nuclear ribonucleoprotein C (*HNRNPC*) is an abundant nuclear RNA-binding protein responsible for pre-mRNA processing (34, 35). *HNRNPC* has been reported to have various functions, such as increasing differentiation in type II testicular germ cell tumors (GCTs), inducing cell death in ovarian cancer, promoting chemoresistance, and indicating OS in gastric cancer and facilitating the progression of colorectal cancer (36–39). *HNRNPC* was found to be a m6A methylation regulator by Liu et al. (40). Meanwhile, the article showed that KD-HNRNPC could up regulate many other genes like MAP3K3 and MTF2 which were reported to be associated with a bad prognosis, respectively, in Hepatocellular Carcinoma and ovarian carcinoma and downregulated other genes like DNAJA3 which was proved to be associated with the increasing of overall survival in breast cancer (41–43). These conclusions implied that the expression of HNRNPC might promote overall survival rate. Besides, the research revealed that low expression of ROBO1 was exhibited worse prognosis in breast cancer patients, which was similar to our finding (44). These studies demonstrated that the possibility that the expression of HNRNPC might be related to a well-prognosis by affecting other genes. Alterations in m6A regulators lead to cancer pathogenesis and development by regulating the expression of tumor-related genes (45).

Therefore, *HNRNPC* might play a role in GBM by regulating other genes that might influence prognosis. The trend of the expression level of *YTHDF2* is not significant observed. However, YTHDF2 has been discovered in prostate cancer which can promote cell proliferation and migration (46). These findings suggested that the up- or down-regulation of specific RNA m6A methylation regulators is linked to cancer development and progression, and the same regulators might have distinct functions in different cancers. Finally, upon combining the results of the univariate Cox and LASSO regression analyses, we observed a similar scenario, which demonstrated that *HNRNPC* had a strong correlation with OS in GBM.

Until now, *HNRNPC* has been reported to have various functions in several kinds of cancer. Additionally, our study showed differences in *HNRNPC* expression by RT-PCR, ICH staining, and WB. *HNRNPC* was upregulated in glioma samples based on RT-PCR and WB analyses, and similar results have been presented in glioblastoma by Park et al. (47). We also observed the OS in the glioma which showed that the high expression of *HNRNPC* might have a good prognosis by Kaplan–Meier method. The result was consistent to our prediction in TCGA dataset. There is a lack of IHC evidence showing *HNRNPC* expression at the protein level in glioma. Thus, we used 25 different grade of glioma tissues that were evaluated immunohistochemically to further study the relationship between the expression of *HNRNPC* and gliomas in depth. We demonstrated that the expression level of *HNRNPC* significantly contributed to malignant progression of glioma. All the results indicated that *HNRNPC* could be a new therapeutic approach to predict diagnosis and prognosis. However, additional experiments to identify the mechanisms of *HNRNPC* are urgently needed.

## Conclusion

**Figure 8:**
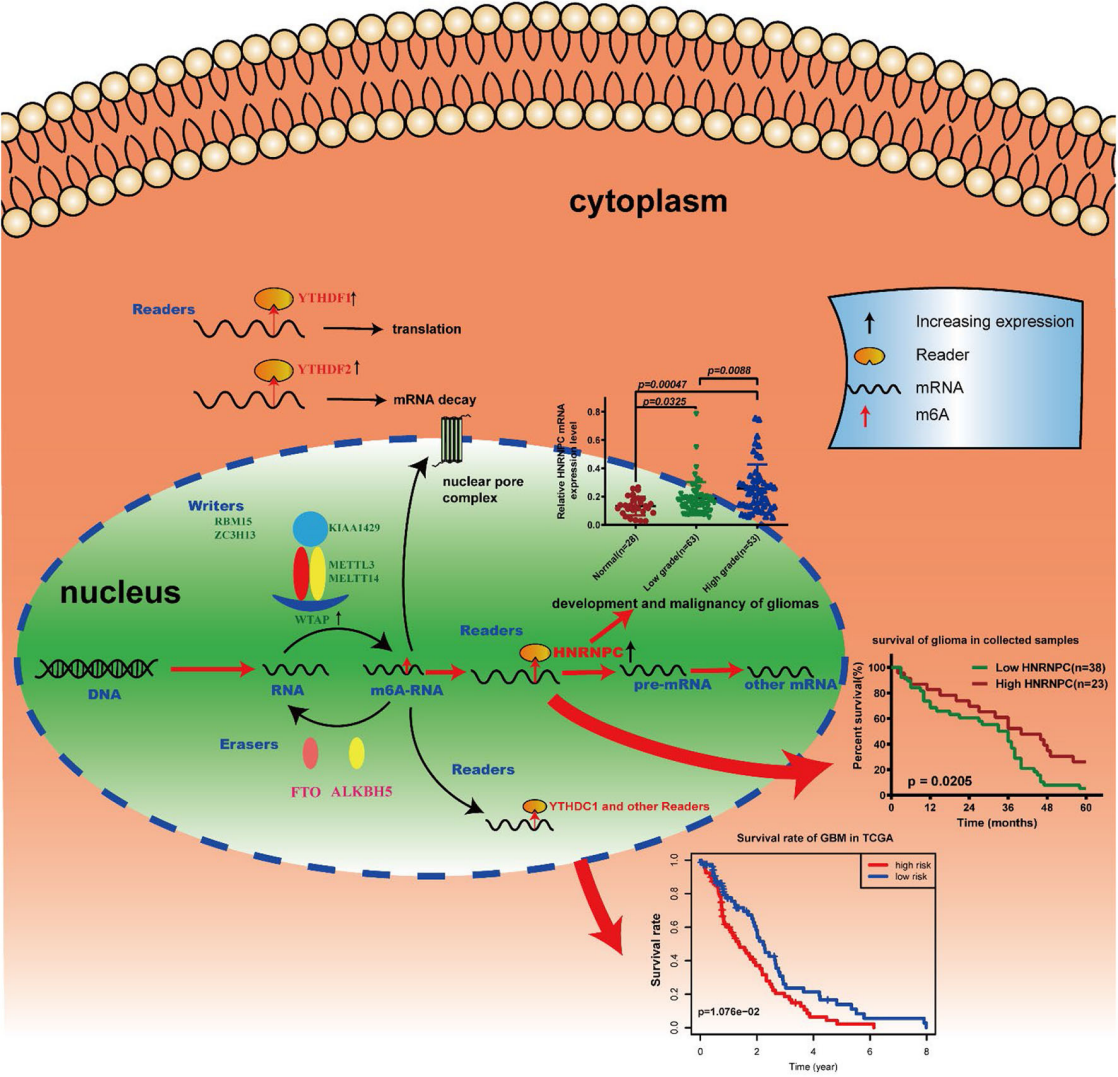
Summary for the expression changes and potential functions of m6A RNA methylation regulators in gliomas.

Our results identified the potential function, prognostic value and expression features of the m6A RNA methylation regulators in GBM and *HNRNPC* could serve as a key biomarker that might be highly associated with the clinical survival rate, m6A methylation levels, and the malignant progression of gliomas (Figure 8). In summary, this study provides a new blueprint for m6A methylation research and important evidence for the future diagnosis and therapy of GBM.

## Data Availability Statement

All datasets generated for this study are included in the article/Supplementary Material.

## Ethics Statement

The studies involving human participants were reviewed and approved by Medical Ethics Committee of the Xiangya Hospital of Central South University (No. 201803806). The patients/participants provided their written informed consent to participate in this study.

## Author Contributions

Q-lL planned and supervised the study. L-cW and S-hC contributed to conception and design, data acquisition, and manuscript drafting. D-cL, Y-lJ, ML, KY, HY, J-JC, C-zQ, M-mL, and Q-xL collected the glioma tissues and clinical information. X-lS and H-yL drafted the article or critically revised it for important intellectual content. All authors read and approved to the final manuscript.

## Conflict of Interest

The authors declare that the research was conducted in the absence of any commercial or financial relationships that could be construed as a potential conflict of interest.
