# Supplementary material for: M6A RNA Methylation Regulator HNRNPC Contributes to Tumorigenesis and Predicts Prognosis in Glioblastoma Multiforme
Source: Front Oncol. 2020 Oct 8;10:536875. doi: 10.3389/fonc.2020.536875 (PMC7578363; doi:10.3389/fonc.2020.536875)
Supplement: Supplementary file 2 [file Data_Sheet_2.docx]

Supplementary Material

Table 1S





Table 1S KEGG pathway enrichment of thirteen m6A regulators.

Figure 1S


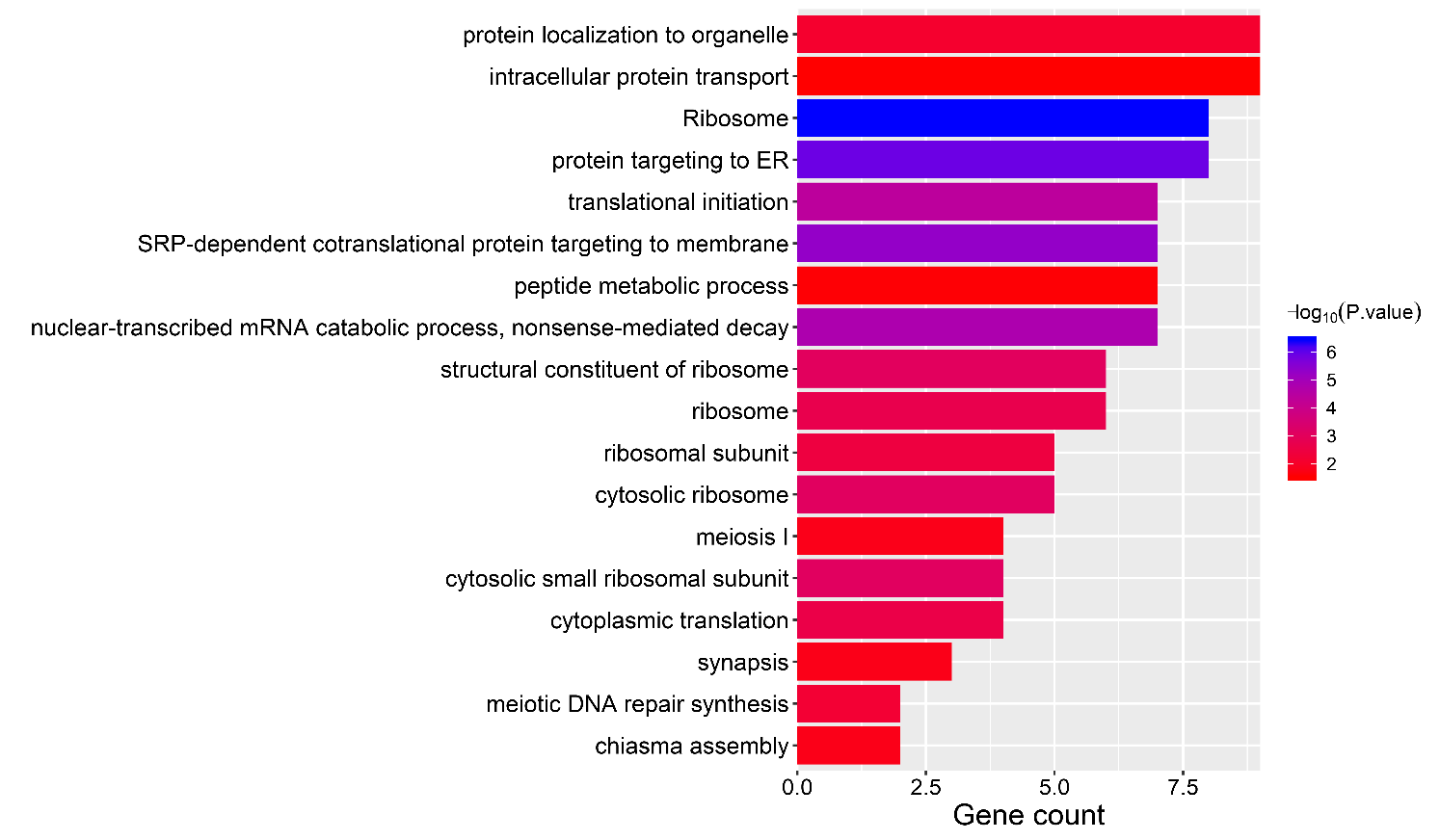


Figure 1S Functional annotation of differential expression of GBM in the two subgroups, Functional annotation of the genes with higher expression in the cluster2 subgroup using GO terms of biological processes cellular component molecular function and KEGG pathway.
